# Nonsurgical molding of congenital auricular deformities and analysis of the correction outcomes: A single-center, retrospective study in east China

**DOI:** 10.3389/fped.2022.1031575

**Published:** 2022-12-16

**Authors:** Chuanbo Liu, Peibin Wo, Jufang Zhang, Jinsheng Li

**Affiliations:** Department of Plastic and Cosmetic Surgery, Affiliated Hangzhou First People's Hospital, Zhejiang University School of Medicine, Hangzhou, China

**Keywords:** nonsurgical molding, congenital auricular deformities, prognostic analysis, retrospective study, east China

## Abstract

**Objective:**

Our research was carried out to provide a clinical reference for the application of nonsurgical therapy in newborns with congenital auricular deformities in east China.

**Methods:**

A retrospective study of consecutive newborns using noninvasive ear molding was conducted in Hangzhou in east China's Zhejiang Province. The demographic and clinical information and photographs of the ear before and after treatment were taken. The diagnosis of each auricular deformity was identified, and the treatment outcome was evaluated.

**Results:**

A total of 224 patients including 356 congenital ear anomalies received noninvasive ear molding. The median age of infants to initiate treatment was 39.5 days. The median treatment duration was 42.5 days. The median follow-up time was 137.0 days. The overall treatment effective rate of all infants with nonoperative ear molding was 92.1%, and mild skin irritation and ulceration occurred in 34 ear deformities (9.6%). It confirmed that the treatment efficiency was satisfactory and the complication rate was still acceptable despite the late initiation treatment of neonates in east China. Further analysis of treatment outcomes among three subgroups of infants (the ages to initiate the ear molding were respectively less than or equal to 28, 29–56, and more than 57 days) revealed that initiation treatment was significantly related to the treatment results and the earlier the initiation treatment, the higher the effective rate and the lower the complication incidence.

**Conclusion:**

Our study hints that newborns in east China may have a longer period for correction. What is more, although our study affirmed a longer period for noninvasive molding, early diagnosis and treatment are still recommended to improve therapy efficiency and reduce treatment duration and complications.

## Introduction

Congenital auricular anomalies are categorized into two major types, namely, deformations and malformations. Deformations are featured with a completely developed pinna with abnormal shape, while malformations are characterized by an underdeveloped auricle owing to partial deficiency of skin and/or cartilage (1, 2). These deformities make children and adults more vulnerable to teasing and bullying by peers, which exerts a negative impact on mental health and social activity (3). As a classical protocol for these deformities, the surgical correction is usually performed at 5 or 6 years of age when the auricle has nearly completed its development and approximates the adult size (4–6). However, surgery may occasionally lead to unnatural contours, subcutaneous hematoma, and residual deformities, and some complications are even more difficult to deal with (5).

Owing to the relatively higher level of circulating estrogen derived from the mother and consequently transient flexibility of auricular cartilage during the first 6 weeks of newborns, ear splinting can exert forces on the pinna to correct most infant deformations and certain malformations (7). Nonsurgical ear molding has been confirmed as a simple, safe, and effective solution since its debut in the 1980s (8, 9). The correction device of molding has evolved from simple raw materials like tapes, glues, and stents to a rigid correction kit such as the EarWell correction system (2, 8–13). This technique is superior to surgery because it can recreate a more natural-looking auricle with better esthetic subunits and fewer residual deformities (1, 14). The concomitant complications are mainly limited to minor skin lesions, most of which can heal in a few days (3, 11). What is more, ear molding is conducted earlier in the neonatal period to avoid infant psychological illness and parental anxiety (3, 14). Recent studies have shown that merely a minority of newborns with auricular deformities will correct spontaneously and the morbidity of the prominent ear will become even higher with auricle development (15). It is impossible for clinicians to predict whether the current deformity will persist, improve, or worsen (7, 9). Many research studies have confirmed that the sooner the molding initiates, the better the outcomes are (7, 10, 12, 13, 15–18). Early ear molding of auricular anomalies not only shortens the therapy duration but also reduces the need for further surgical correction.

The noninvasive ear correction concept and system is not universally applied in China, and articles on molding in infants with ear deformities in east China are rare, although it has been brought into China for several years. From our clinical experience, the initiation treatment time is comparatively late compared with that for newborns in developed countries. This may be related to the misconception held by many parents, obstetricians, and pediatricians that ear deformity will self-correct with time. Actually, up to one-third of ear anomalies will correct themselves (1, 2), and thus, many children may lose the valuable opportunity for early noninvasive correction. Given that the population of newborns in China is large and the incidence of ear deformities is up to 57.5% (2), early nonsurgical correction will provide more benefits to newborns with congenital auricular anomalies.

Therefore, we illustrated the demographic characteristics of newborns with auricular deformities in our plastic and esthetic center in Hangzhou in east China and analyzed the treatment outcomes and its influencing factors to provide clinical experience in correcting ear anomalies with ear molding systems in the future.

## Patients and methods

### Patients and protocol

A single-center-based, retrospective study of consecutive newborns with congenital auricular deformities using a nonsurgical ear molding system was conducted between 2019 and 2021 in the Plastic and Cosmetic Surgery Department of Affiliated Hangzhou First People's Hospital, Zhejiang University School of Medicine. All infants included in our study were younger than 6 months. The newborns with microtia, hearing dysfunction, or other systemic diseases such as congenital heart diseases were excluded. The plasticity of ear cartilage was examined with digital pressure and ensured that the aberrant ears were liable to be corrected with noninvasive molding. If parents planned to correct the infant's auricle deformities with nonoperative ear molding, the benefits and possible risks were explained in detail and then informed consent was signed. It was difficult to obtain symmetry between the bilateral ears, and parents were particularly told that the main purpose of therapy was to remold the normal auricle appearance. The study was reviewed by the ethics committee of Affiliated Hangzhou First People's Hospital, Zhejiang University School of Medicine.

According to Professor Byrd's classification system (19), varieties of deformations included in our study were categorized as prominent ear, lidding ear, helical rim abnormality, Stahl's ear, prominent conchal crus, and mixed deformities, while the types of malformations were classified as constricted ear and cryptotia. The diagnosis of auricular deformation or malformation was identified by two chief plastic surgeons. What needs to be distinguished especially were a lidding ear, cup ear, and constricted ear. The folding over of the helical rim and scapha without a shortage of skin and cartilage was a lidding ear, while helical folding secondary to a deficit of skin and cartilage with varying degrees was a constricted ear, and the cup ear was regarded as a special type of constricted ear.

The photographs of the ear before and after treatment were taken and documented. Demographic and clinical information including the age of initiation treatment, gender, family history of auricular defect, coexisting comorbidity, delivery method, feeding pattern during therapy, and motivation for treatment were obtained through an interview with parents during the first consultation with our clinic. The treatment duration and follow-up time were also calculated at the end of the study. The treatment duration was defined as a therapy period between the initiation time to wear the splinting and the time when the ideal ear appearance was achieved. All children were followed up until they were 6 months old.

The treatment outcome of ear molding was graded as excellent (normal or nearly normal auricle), good (improved but not a normal auricle), or poor (slight or no improvement in ear shape) by the chief plastic surgeon based on the comparison of preoperative and postoperative auricular photographs. If the treatment outcome was assessed as excellent or good, the therapeutic efficacy was considered effective; if the result was rated as poor, the treatment effect was deemed ineffective. Then, the effective rate was obtained. Furthermore, the complications accompanied by treatment including erythema, skin, and/or cartilage ulceration were also recorded.

### Nonsurgical ear correction system and its applications

**Figure 1:**
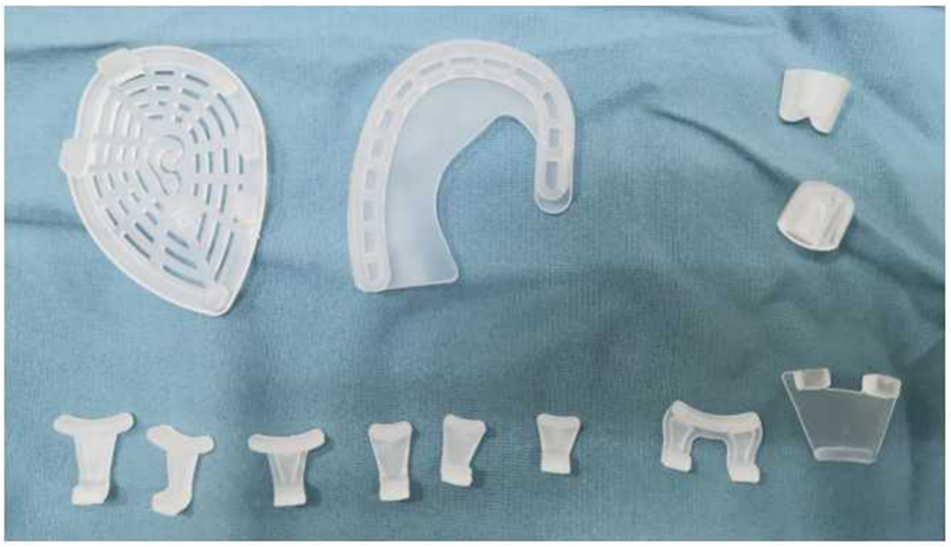
EarLimn Infant Ear Correction system.

The ear molding device used in the study was the EarLimn Infant Ear Correction system (Hunan Cihui Medical Technology Co., Ltd., China), including a base, several stents, several retractors, a conchal conformer, and a cover (Figure 1). All components were made of silicone and assembled to shape the auricle.

The ear correction system was initiated to wear during the first visit to the outpatient and assembled by authors JL and CL. First, the skin surface to place the base around the external auricle was shaved and cleaned. Then, 3M Steri-Strip adhesive was applied to the skin of the scapha and retroauricular sulcus to reduce the pressure from retractors and stents. The auricle base was pasted on the skin around the ear. The stent was located posteriorly along the retroauricular sulcus in the upper third part of the auricle to create the antihelix and superior crus. The retractor was anteriorly rested on the scapha to expand the helical rim and hold the ear in the desired position, and two more strips of adhesive were used to strengthen the fixation of the retractor. The anterior retractor could not overlap the posterior stent to avoid pressure damage on the skin and cartilage. If there was a prominent conchal crus, the conchal former was used to rectify the projecting concha with anteriorly directed forces. The cover was placed on the top to protect the device from scratching by children.

All infants wore the correction device 24 h a day and kept the area around the auricle dry under the good care of their parents. The infants with deformities were advised to re-examine the shape of auricles and adjust the devices 1 week after the initial treatment and then were scheduled to visit our department regularly at a 2-week interval in the follow-up treatment. If parents discover any looseness or displacement of the correction device, they should take infants to reinstall the molding kit in our treatment room timely. Once there was skin sensitivity, eczema, or skin erosion, ear molding was suspended temporarily until skin lesions healed. The simple retention taping was carried on using 3M tape to stabilize the therapeutic effect even when the desired shape was obtained. The clinicians taught the parents how to manipulate the retention molding using 3M adhesive, and simple tape retention was employed throughout the follow-up period until the infant was 6 months old to prevent a relapse.

### Statistical analysis

Statistical analysis was conducted by SPSS 25 for Windows in this study. *P* < 0.05 was considered statistically significant. The statistical descriptions of continuous variables were expressed as mean with standard deviation or median with interquartile range depending on whether the statistical data fitted a normal distribution. The statistical descriptions of categorical variables were presented by number and percentage. Binary logistic regression analysis was adopted to determine the independently influencing factors of the treatment outcome in all ear deformities. The K-independent sample nonparametric test was used to compare differences in the median treatment duration among the three subgroups of ear deformities. Pearson’s chi-square test was used to compare the effective rate and incidence of complications among the three subgroups of ear deformities.

## Results

There were 225 consecutive infants treated with the EarLimn Ear Correction system from November 2019 to November 2021. Only one child experienced an erythematous rash whenever he wore the correction application, and his parents decided to withdraw from the study because of a severe skin allergic reaction. As a result, 224 patients including 356 congenital ear anomalies received noninvasive ear molding and completed follow-up visits until all the patients were 6 months old.

### Demographics of the 224 patients

Of the 224 patients, 135 (60.3%) were boys and 89 (39.7%) were girls. A total of 132 babies (58.9%) had bilateral ear deformities, 41 babies (18.3%) had left ear deformities, and 51 babies (22.8%) had right ear deformities. A total of 207 newborns (92.4%) did not have a familial history of ear deformities, and 17 newborns (7.6%) reported a first-degree relative with ear deformities. A total of 215 infants (96.0%) did not have any coexisting comorbidity, while 9 infants (4%) had slightly concomitant deformities such as hand and/or foot deformity. One hundred fifty-eight neonates (70.5%) were delivered vaginally, and 66 neonates (29.5%) were delivered by a cesarean section. One hundred ninety-seven babies (87.9%) were fed by breast, 7 babies (3.1%) were fed by formula, and 20 babies (8.9%) were fed by breast and formula during the treatment period. Forty-six patients (20.5%) were self-motivated by their parents to initiate ear molding to address the ear deformity, and 178 patients (79.5%) conducted therapy owing to the doctor's advice.

The detailed data of demographics in 224 patients are demonstrated in Table 1.

**Table 1:** Demographics of the 224 patients.

Category	Number (%)
Number of patients	224 (100)
Number of abnormal ears	356 (100)
Gender	
Male	135 (60.3)
Female	89 (39.7)
Abnormal auricle appearance	
Bilateral ears	132 (58.9)
Left ear	41 (18.3)
Right ear	51 (22.8)
Family history of auricular defect	
Yes	17 (7.6)
No	207 (92.4)
Coexisting comorbidity	
Yes	9 (4)
No	215 (96.0)
Delivery method	
Vaginal	158 (70.5)
Cesarean birth	66 (29.5)
Feeding pattern	
Breastfeeding	197 (87.9)
Formula feeding	7 (3.1)
Mixed feeding	20 (8.9)
Motivation of treatment	
Self-motivated by parents	46 (20.5)
Doctor's advice	178 (79.5)

### Outline of 356 neonatal ear deformities

Three hundred fifty-six abnormal ears of 224 patients were classified into eight types of congenital ear deformities, including prominent ear (39 ears, 11.0%), lidding ear (74 ears, 20.8%), helical rim abnormality (111 ears, 31.2%), Stahl's ear (13 ears, 3.7%), prominent conchal crus (7 ears, 2.0%), mixed deformities (8 ears, 2.2%), cryptotia (20 ears, 5.6%), and constricted ear (84 ears, 23.6%).

**Figure 2:**
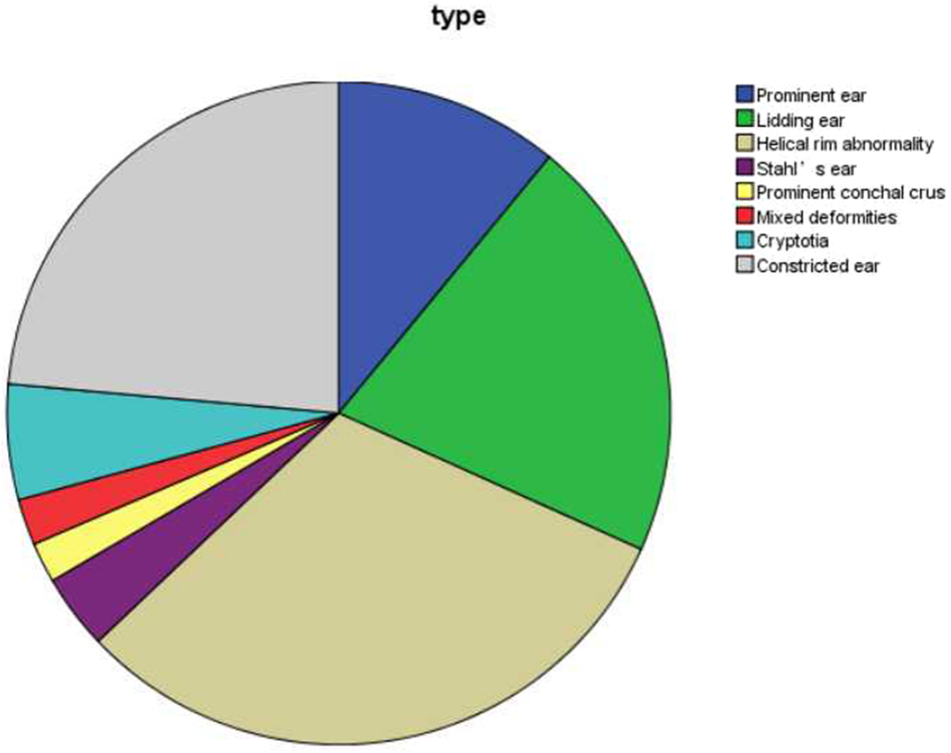
Type of congenital ear deformities in 356 ears.

The number and percentage of eight different kinds of congenital ear deformities in our study are exhibited in Figure 2.

### Treatment outcome and analysis of its influencing factors in all infants

**Figure 3:**
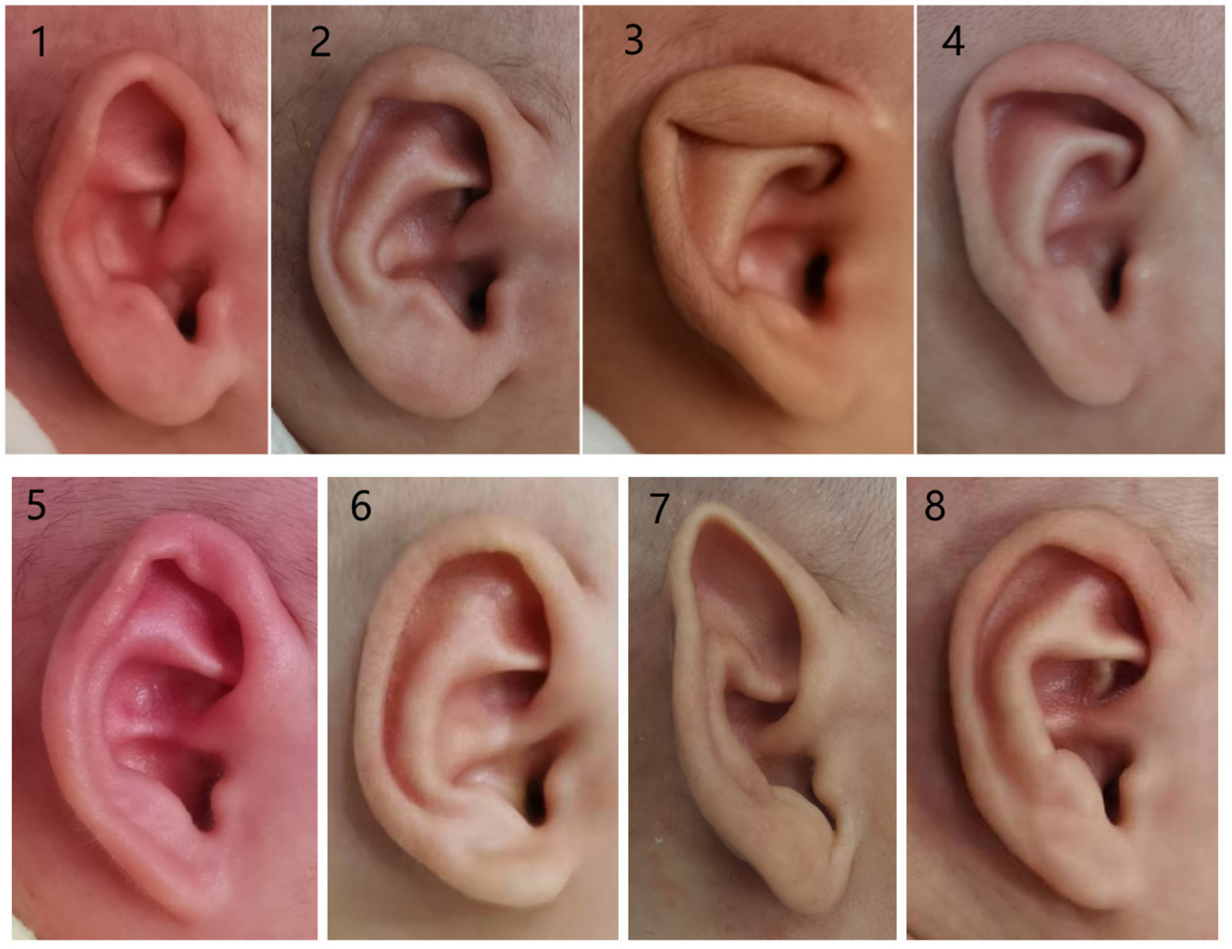
Photographs of congenital ear deformities before and after treatment (part I): (1 and 2) before and after treatment of the prominent ear, (3 and 4) before and after treatment of the lidding ear, (5 and 6) before and after treatment of the helical rim abnormality, and (7 and 8) before and after treatment of Stahl's ear.

**Figure 4:**
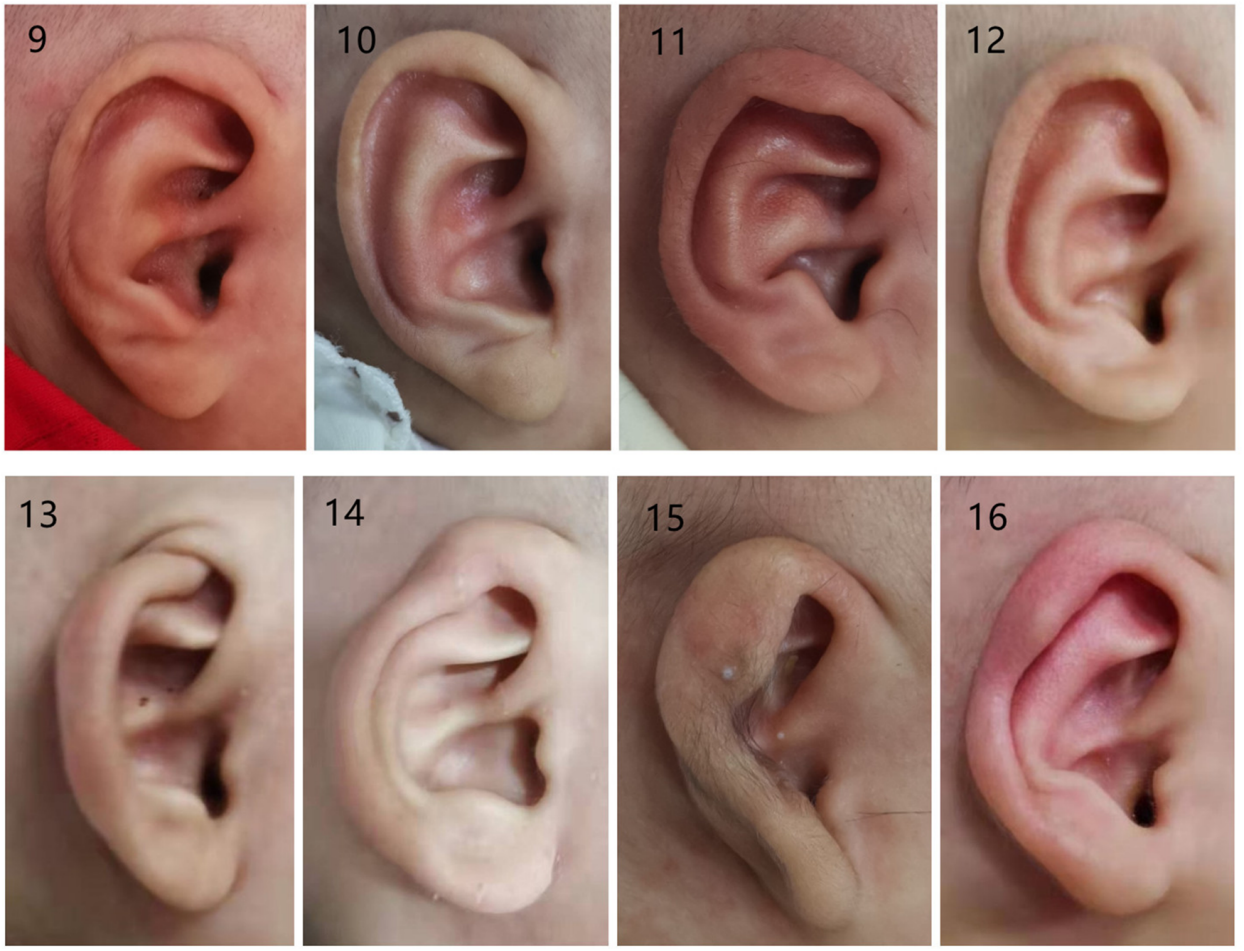
Photographs of congenital ear deformities before and after treatment (part II): (9 and 10) before and after treatment of the prominent conchal crus, (11 and 12) before and after treatment of mixed deformities, (13 and 14) before and after treatment of the cryptotia, (15 and 16) before and after treatment of the constricted ear.

The typical auricular shapes of eight different kinds of congenital ear deformities before and after molding therapy are displayed in Figures 3, 4.

**Figure 5:**
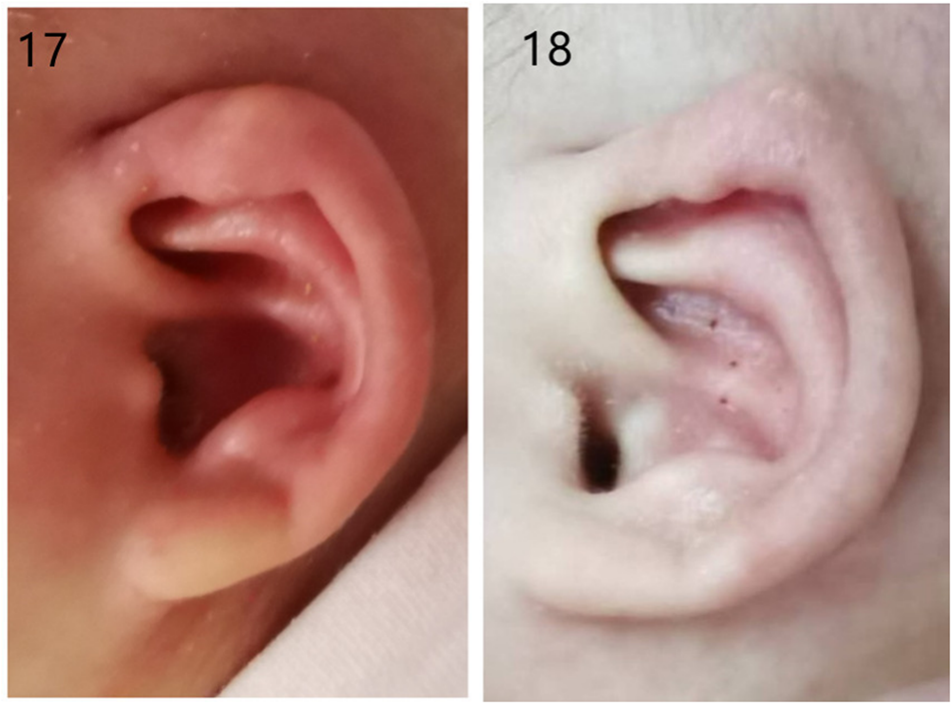
Photographs of failure therapy before and after treatment: (17 and 18) before and after treatment of 4-month-old neonates.

The failure pictures before and after treatment in a 4-month-old baby are shown in Figure 5.

The age of infants with auricular deformities to initiate treatment ranged from 3 to 161 days, and the median age was 39.5 days. The treatment duration was between 7 and 150 days, and the median treatment duration was 42.5 days. The median follow-up time was 137.0 days.

The correction outcomes of congenital ear deformities were evaluated as excellent in 168 ears (47.2%), good in 160 ears (44.9%), and poor in 28 ears (7.9%). The therapeutic efficacy (excellent and good outcomes) was considered effective in 328 cases, and the overall treatment effective rate of nonoperative molding was 92.1%.

Mild skin irritation and ulceration occurred in 34 ear deformities (9.6%). Once the complications occurred, the treatment was suspended and topical antibiotic ointment was applied to the wound. The correction devices were installed again after the skin injuries were cured.

The related indicators of treatment outcomes in 356 ear deformities are shown in Table 2.

**Table 2:** Treatment outcomes of 356 ear deformities.

Categories	Values
Age of treatment initiation (days)	39.5 (16–49)
Treatment duration (days)	42.5 (30–55)
Follow-up time (days)	137 (125–150)
Correction outcome, number (%)	
Excellent	168 (47.2)
Good	160 (44.9)
Poor	28 (7.9)
Therapeutic efficacy, number (%)	
Effective	328 (92.1)
Ineffective	28 (7.9)
Complication, number (%)	
Yes	34 (9.6)
No	322 (90.4)

Logistic regression analysis revealed that feeding pattern (*P* = 0.000) and age of initiation treatment (*P* = 0.001) were the two significant influencing factors of the treatment outcome. Gender, type of ear deformity, and treatment duration did not impact the therapy result (*P* > 0.05).

The results of logistics regression are displayed in Table 3.

**Table 3:** Factors related to the treatment outcome.

	B	SE	Wald	df	Sig.	Exp (B)	95.0% CI for Exp (B)	
							Lower	Upper
Feeding pattern	1.529	0.256	35.590	1	0.000	4.616	2.793	7.629
Age of treatment initiation	0.026	0.007	12.005	1	0.001	1.026	1.011	1.041

### Comparison of treatment outcomes among different subgroups of infants

The median age to start therapy was 39.5 days, which was later in contrast with the initiation treatment time of other study populations abroad. According to the characteristics of our data distribution, all patients were allocated to three subgroups. The ages to initiate ear molding in the three groups were respectively less than or equal to 28, 29–56, and more than 57 days.

The median treatment duration among the three groups was significantly different (28 vs. 48 vs. 70 days, *P* < 0.05). The incidence of complications was significantly different (6.2% vs. 6.5% vs. 26.3%, *P* < 0.05). The effective rate was significantly different (97.2% vs. 94.8% vs. 71.9%, *P* < 0.05).

The results of the comparison among three subgroups are revealed in Table 4.

**Table 4:** Comparison of treatment outcomes among different subgroups of infants.

Group	Age of treatment initiation (days)	Ear deformities, number (%)	Treatment duration (days)	Therapeutic efficacy, number (%)		Complications, number (%)	
				Effective	Ineffective	Yes	No
1	≤28	145 (40.7)	28 (20–35)	141 (97.2)	4 (2.8)	9 (6.2)	136 (93.8)
2	28–56	154 (43.3)	48 (42–56)	146 (94.8)	8 (5.2)	10 (6.5)	144 (93.5)
3	>57	57 (16.0)	70 (55–80)	41 (71.9)	16 (28.1)	15 (26.3)	42 (73.7)

These results among the three groups demonstrated that the later the initiation treatment started, the worse the effective rate (97.2% vs. 94.8% vs. 71.9%, *P* < 0.05). With the delay in initiation treatment, the median treatment duration increased (28 vs. 48 vs. 70 days, *P* < 0.05), and the incidence of complications was higher (6.2% vs. 6.5% vs. 26.3%, *P* < 0.05).

## Discussion

Nonsurgical molding of congenital auricular deformities has been widely accepted in developed countries for several decades since it was first introduced in the 1980s in Japan and America (8, 9, 16). The incidence of newborn congenital auricular deformities in south China was as high as 57.47% (20). Several domestic auricular correction devices, except for the EarWell Correction system, are available in China in recent years (21). However, a few clinical research studies published in English journals can be found because the noninvasive therapy introduced into the Chinese mainland is comparatively later (11, 15, 21). To provide some reference for nonsurgical therapy of newborn auricular deformities in east China, our research thus analyzed the nonsurgical treatment outcome and its influencing factors in infants using a domestic correction system. Our study confirmed that the treatment efficiency (92.1%) was satisfactory and the complication rate (9.6%) was still acceptable despite the late initiation treatment of neonates (the median age of infants to initiate treatment was 39.5 days) for all infants with nonoperative ear molding. Further analysis of treatment outcomes among three subgroups of infants (the ages to initiate the ear molding were respectively less than or equal to 28, 29–56, and more than 57 days) revealed that initiation treatment was significantly related to the treatment results, and the earlier the initiation treatment, the higher the effective rate and the lower the complication incidence.

Our research showed that the overall treatment effective rate for all infants with nonoperative ear molding was 92.1%, and mild skin irritation and ulceration occurred in 34 ear deformities (9.6%). A comparison with the results of other previous studies (1, 7, 17, 22) indicated that the treatment efficiency was comparable with results of other studies and the complication rate was not elevated despite late initiation treatment of neonates in east China. For the moment, the upper age limit of infants, namely, the time window, is still in dispute (23), although treatment initiation of nonsurgical correction as early as possible is crucial to achieving a satisfactory outcome. A growing number of American and European studies emphasize the importance of early treatment timing (1, 2, 7, 9, 13, 16–18). Their results reveal that the correction outcome is effective only when ear molding is initiated within the first few days or weeks of age and delayed treatments usually result in less favorable consequences. However, some other research studies report that older children can also obtain good correction results if the initial treatment is extended up to 6 months, even 5 or 14 years of life (8, 24–26). Our results hint that the newborn population in east China may have a longer time window for correction. It is consistent with the conclusions obtained in the Japanese population (8, 25, 26), and it might be related to racial differences. We suspect that the elasticity of ear cartilage in the infant population in east China may change more slowly over time than that in the Western population, and the ear is more flexible to be corrected. However, there is lack of a useful method or tool to assess the flexibility of auricular cartilage objectively. What is more, maternal estrogen levels in breastfed babies remain at higher levels even after 6 weeks of age, and this increases cartilage flexibility (21). This good result may also be related to the tape retention treatment following the wear of a noninvasive correction system. All of our patients did not stop treatment immediately after they achieved favorable auricle morphology and used 3M tape to maintain the ideal appearance until they were 6 months old.

The significant factors affecting the treatment effectiveness in our study were the age of initiation treatment and feeding pattern. Further analysis of subgroups at different times to start treatment manifested that the earlier the initiation of treatment, the higher the effective rate and the lower the complication incidence. The relatively late initiation of treatment may be one of the potential reasons to result in poor treatment outcomes. As a result, although our study affirmed a longer time window for noninvasive correction, early initiation treatment is still recommended to improve therapy efficiency and reduce the treatment duration and complications. First, multidisciplinary cooperation among plastic surgeons, pediatricians, and otolaryngologists should be strengthened to promote early screening, diagnosis, and treatment (22). Second, relatively expensive spending on the correction system cannot be covered by medical insurance in China. Some parents may give up treatment because of the total treatment cost in poor areas. The health department is supposed to propel the inclusion of treatment expenditure into the national medical security system and break down therapy barriers caused by cost. What is more, several simpler and higher cost-effective correction techniques (27, 28) can also be introduced to reduce the cost of treatment. Feeding patterns had an impact on the treatment outcome, and breastfeeding led to superior outcomes (21). The levels of circulating estrogen decline distinctly after 6 weeks, but breastfeeding increases maternal estrogen levels in older children. Subsequently, flexible ear cartilage has a more sensitive response to nonsurgical molding (29).

To get better results in the treatment of newborns with congenital auricular deformities in the future, we profoundly figure out the social factors that lead to the relatively late initiation of treatment. The parents and medical staff concentrate mainly on the hearing function of the ear and disregard the abnormal auricular morphology. Most pediatricians and otolaryngologists have a false understanding that auricular deformity can be improved or corrected without therapy during auricle development. In fact, only about 30% of congenital auricular deformities can self-heal without intervention (1, 20). Furthermore, the noninvasive correction treatment has not been extensively applied in China, and the parents do not know when and how to seek nonoperative remedies. The vast majority of infants with ear abnormalities therefore miss the best treatment opportunity and undergo surgical intervention until they are 6 years old. These negative factors should be addressed to detect and treat auricular deformities as early as possible. To minimize the occurrence of complications, the parents were required to take newborns wearing ear molding to our department at least every 2 weeks to observe the treatment outcome and adjust the device in time. The parents were also educated to observe closely whether the correction device was loose or displaced because this would lead to skin injury owing to the increased pressure on the skin. Once skin lesions were detected, the treatment was suspended instantly and topical antibiotic ointment was applied. The therapy was continued following the healing of skin lesions. In addition, ear molding should be initiated as soon as possible to reduce the rate of complications based on the conclusions of our study.

There were several limitations of our study. First, the design of the retrospective study was its major disadvantage. To minimize the result bias of our research, the diagnosis, therapy, and evaluation were performed by experienced surgeons. The demographic and clinical information was also obtained and recorded accurately. Second, all the infants with congenital auricular deformities were recruited from one plastic surgery center. The results would be more persuasive if the study recruited more individuals from multiple medical centers across China. Third, there is a lack of defined diagnostic criteria for each specific type of auricular deformity until now; therefore, it is difficult to strictly distinguish some abnormalities such as prominent ear, cup ear, and lidding ear according to the current classification standards (19). Even the same research team adopted different classification methods in articles of different periods (1, 19). Perhaps this is a potent reason why the type of ear deformity did not affect the treatment outcomes in this study. In our research, the accurate diagnosis and classification criteria of congenital ear deformities, particularly the definition of constricted ear and lidding ear, were adopted to make the results more reliable.

## Conclusion

On the one hand, the treatment efficiency (92.1%) was satisfactory and the complication rate (9.6%) was still acceptable, although the median age of infants to initiate treatment was 39.5 days for all infants with nonoperative ear molding. It indicated that good treatment outcomes in east Chinese infants could be obtained despite relatively late initiation treatment of ear molding. On the other hand, further analysis of treatment outcomes among three subgroups of infants at different ages to initiate ear molding demonstrated that initiation treatment was significantly related to the treatment results, and the earlier the initiation treatment, the higher the effective rate and the lower the complication incidence. In conclusion, although our study affirmed a longer time window for noninvasive molding, early diagnosis and treatment by multidisciplinary cooperation are still recommended to improve therapy efficiency and reduce treatment duration and complications. Also, more multiple-center, prospective studies with long-term follow-up should be conducted to draw more reliable conclusions in infants with ear deformities in east China.

## Data Availability

The raw data supporting the conclusions of this article will be made available by the authors, without undue reservation.
